# A review of methods used to kill laboratory rodents: issues and opportunities

**DOI:** 10.1177/00236772221097472

**Published:** 2022-05-25

**Authors:** Jasmine M Clarkson, Jessica E Martin, Dorothy E F McKeegan

**Affiliations:** 1 Institute of Biodiversity, Animal Health and Comparative Medicine, University of Glasgow, UK; 2 The Royal (Dick) School of Veterinary Studies and The Roslin Institute, The University of Edinburgh, UK

**Keywords:** Animal welfare, ethics, euthanasia methods, mice, rats, 3Rs

## Abstract

Rodents are the most widely used species for scientific purposes. A critical pre-requisite of their use, based on utilitarian ethical reasoning, is the provision of a humane death when necessary for scientific or welfare grounds. Focussing on the welfare challenges presented by current methods, we critically evaluate the literature, consider emerging methodologies that may have potential for refinement and highlight knowledge gaps for future research. The evidence supports the conclusion that scientists and laboratory personnel should seek to avoid killing laboratory rodents by exposing them to carbon dioxide (CO_2_), unless exploiting its high-throughput advantage. We suggest that stakeholders and policymakers should advocate for the removal of CO_2_ from existing guidelines, instead making its use conditionally acceptable with justification for additional rationale for its application. With regards to physical methods such as cervical dislocation, decapitation and concussion, major welfare concerns are based on potential inaccuracy in application and their susceptibility to high failure rates. There is a need for independent quality-controlled training programmes to facilitate optimal success rates and the development of specialist tools to improve outcomes and reliability. Furthermore, we highlight questions surrounding the inconsistent inclusion criteria and acceptability of physical methods in international regulation and/or guidance, demonstrating a lack of cohesion across countries and lack of a comprehensive ‘gold standard’ methodology. We encourage better review of new data and championing of open access scientific resources to advocate for best practice and enable significant changes to policy and legislation to improve the welfare of laboratory rodents at killing.

## Examen des méthodes utilisées pour euthanasier les rongeurs de laboratoire: questions et possibilités

Les rongeurs sont les espèces les plus utilisées à des fins scientifiques. Une euthanasie humaine, lorsque nécessaire pour des raisons scientifiques ou de bien-être, constitue une condition préalable critique de leur utilisation, fondée sur un raisonnement éthique utilitaire. En nous concentrant sur les défis de bien-être présentés par les méthodes actuelles, nous évaluons de façon critique la littérature et considérons les nouvelles méthodologies qui pourraient avoir un potentiel de raffinement. Nous soulignons également les lacunes de connaissances pour la recherche future. Les preuves permettent de conclure que les scientifiques et le personnel de laboratoire devraient chercher à éviter l’euthanasie des rongeurs de laboratoire en les exposant au dioxyde de carbone (CO_2_), à moins d'exploiter son avantage à haut rendement. Nous suggérons que les parties prenantes et les décideurs politiques préconisent la suppression du CO_2_ des lignes directrices existantes, plutôt que de rendre son utilisation conditionnellement acceptable, ce qui constitue une justification supplémentaire de son application. En ce qui concerne les méthodes physiques telles que la luxation cervicale, la décapitation et la commotion cérébrale, les préoccupations majeures de bien-être sont basées sur l’inexactitude potentielle dans l’application et leur susceptibilité à entraîner des taux élevés d'échec. Il est nécessaire de mettre en place des programmes de formation indépendants et contrôlés par la qualité afin de faciliter des taux de réussite optimaux et de développer des outils spécialisés pour améliorer les résultats et la fiabilité. Nous soulignons en outre les questions entourant les critères d’inclusion incohérents et l’acceptabilité des méthodes physiques dans la réglementation et/ou les directives internationales, démontrant un manque de cohésion entre les pays et l’absence d’une méthodologie complète de référence. Nous encourageons un meilleur examen des nouvelles données et la promotion des ressources scientifiques à accès libre pour préconiser les meilleures pratiques et permettre d’apporter d’importants changements aux politiques et à la législation afin d’améliorer le bien-être des rongeurs de laboratoire lors de leur euthanasie.

## Eine Untersuchung von Methoden zur Tötung von Labornagern – Probleme und Chancen

Nagetiere sind die am häufigsten für wissenschaftliche Zwecke verwendete Tierart. Eine wesentliche Voraussetzung für ihre Verwendung, die auf utilitaristischen ethischen Überlegungen beruht, ist die Gewährleistung eines humanen Todes, wenn dies aus wissenschaftlichen oder tierschutzrechtlichen Gründen erforderlich ist. Unter besonderer Beachtung der Probleme des Tierschutzes bei den derzeitigen Methoden werten wir die Literatur kritisch aus, betrachten neue Methoden mit Verbesserungspotenzial und zeigen Wissenslücken auf, die im Rahmen künftiger Forschung zu berücksichtigen sind. Die gewonnenen Erkenntnisse stützen die Schlussfolgerung, dass Wissenschaftler und Labormitarbeiter versuchen sollten, die Tötung von Labornagern durch Kohlendioxid (CO_2_) zu vermeiden, es sei denn, sie nutzen seinen Hochdurchsatz-Vorteil aus. Wir empfehlen Beteiligten und politischen Entscheidungsträgern dafür zu plädieren, dass der Einsatz von CO_2_ aus den bestehenden Leitlinien gestrichen und stattdessen unter Vorbehalt und Anführung zusätzlicher Gründe zur Rechtfertigung der Verwendung akzeptiert wird. Bei physikalischen Methoden wie zervikale Dislokation, Dekapitation und Gehirnerschütterung bestehen große Bedenken hinsichtlich des Wohlergehens der Tiere aufgrund potenziell inkorrekter Anwendung und hohen Versagensquoten. Es besteht ein Bedarf an unabhängigen, qualitätskontrollierten Schulungsprogrammen zur Gewährleistung optimaler Erfolgsquoten und an der Entwicklung spezieller Instrumente zur Verbesserung von Ergebnissen und Zuverlässigkeit. Darüber hinaus werfen wir Fragen im Zusammenhang mit den uneinheitlichen Auswahlkriterien und der Akzeptanz physikalischer Methoden in internationalen Vorschriften und/oder Leitlinien auf, die einen Mangel an Kohärenz zwischen den Ländern und das Fehlen einer umfassenden „Goldstandard“-Methodik belegen. Wir regen eine gründlichere Analyse neuer Daten und die Nutzung frei zugänglicher wissenschaftlicher Ressourcen an, um Best Practice durchzusetzen und grundlegende Änderungen in Politik und Gesetzgebung mit dem Ziel eines verbesserten Tierschutzes bei der Euthanasie von Labornagern zu ermöglichen.

## Una revisión de los métodos utilizados para el sacrificio de roedores de laboratorio: problemas y oportunidades

Los roedores son la especie más utilizada con fines científicos. Un prerrequisito crítico de su uso, basado en un razonamiento ético utilitario, es sacrificarlos de una forma humana cuando sea necesario por motivos científicos o de bienestar. Centrándonos en los problemas de bienestar que presentan los métodos actuales, evaluamos críticamente las publicaciones pertinentes, consideramos las metodologías emergentes que pueden tener potencial para ser refinadas y destacamos las lagunas de conocimiento para futuras investigaciones. Las pruebas respaldan la conclusión de que los científicos y el personal de laboratorio deberían evitar sacrificar a los roedores de laboratorio mediante la exposición al dióxido de carbono (CO_2_), a menos que aprovechen su ventaja de alto rendimiento. Sugerimos que las partes interesadas y los responsables políticos aboguen por la eliminación del CO_2_ de las directrices existentes, y que en su lugar hagan que su uso sea aceptable de forma condicional con la justificación de razones adicionales para su aplicación. En lo que respecta a los métodos físicos como la dislocación cervical, la decapitación y la conmoción cerebral, las principales preocupaciones en materia de bienestar se basan en la posible inexactitud en la aplicación y su susceptibilidad a un alto porcentaje de error. Se necesitan programas de formación independientes y de calidad controlada para facilitar unos índices de éxito óptimos y el desarrollo de herramientas especializadas para mejorar los resultados y la fiabilidad. Asimismo, destacamos las cuestiones relacionadas con la inconsistencia de los criterios de inclusión y la aceptabilidad de los métodos físicos en la regulación y/o la orientación internacional, lo que demuestra una falta de cohesión entre los países y la falta de una metodología integral de “referencia principal”. Recomendamos una mejor revisión de los nuevos datos y la defensa de los recursos científicos de acceso abierto para abogar por mejores prácticas y permitir cambios significativos en la política y la legislación a fin de mejorar el bienestar de los roedores de laboratorio a la hora de sacrificarlos.

## Why consider killing methods for use in laboratory rodents?

Rodents remain the most widely used species for scientific research due to their small size, low cost, rapid sexual maturity and scope for genetic manipulation. Despite significant interest in the replacement of animals for scientific purposes,^1,2^ the numbers involved are still large, and growing, primarily due to advances in molecular genetics.^3^ In 2019, global animal use for scientific research was estimated to be 192.1 million annually.^4^ In Europe (and Norway), approximately 6.4 million mice and rats were used in 2018 (most recent published data), accounting for around 62% of the animals reported across member states of the European Union (EU).^5^ Therefore, global numbers are likely to be substantial, potentially exceeding 100 million rats and mice annually.

Regulation requires animals to be killed humanely upon completion of the work (i.e. at the end of the experiment or breeding programme) and when humane endpoints are reached.^6–9^ This necessitates the killing of millions of laboratory rodents each year. Ethical harms relating to the harm of death notwithstanding,^10^ the scale of this activity makes welfare at the time of killing an important issue. Arguably, identification and use of methods that offer a death with minimal suffering is a moral imperative, and crucial to support the justifiability of animal-based research based on utilitarian harm/benefit ethical reasoning.

There are diverse international requirements for killing methods for laboratory rodents (Table 1), which include: overdose of anaesthetic (with various inhalant or injectable agents), concussion by blunt force trauma, cervical dislocation, decapitation, exposure to carbon dioxide (CO_2_) or carbon monoxide (CO) and microwave irradiation. If animals are unconscious (e.g. anaesthetised), methods such as exsanguination, air embolism and injection of potassium chloride or ethanol are permitted.^11^ Across member states of the EU and in the United Kingdom (UK), killing laboratory rodents is regulated by law; however, in the United States (US), Canada, Australia and New Zealand this is not the case.^6–8^ Instead, approaches are mandated through national guidelines and local policy rather than law, and methods are classified according to whether they are considered capable of providing a ‘humane’ death. Thus, they may be described as acceptable, conditionally acceptable (humane only when done correctly and appropriately and only when operator health and safety concerns are mitigated) or unacceptable. Although not intended to be exhaustive, Table 1 highlights the international significant differences in requirements for rodents at the time of killing. These represent the potential for misalignment of animal care standards and killing practices which could present numerous challenges to animal welfare including unnecessary suffering at the time of killing.

**Table 1.** Overview of killing methods from published euthanasia guidelines for adult laboratory rodents across scientifically advanced countries. Light blue represents methods permitted or recommended for use, dark blue represents methods where additional permissions are required, navy blue represents methods not permitted or recommended.

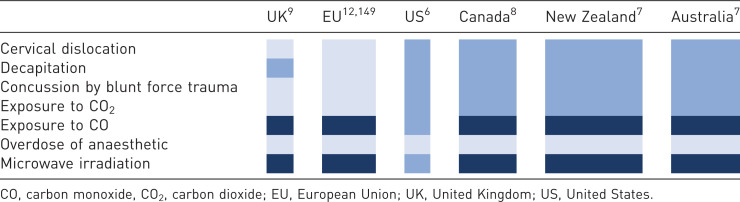

The aim of this review is to critically evaluate the literature to identify the welfare challenges presented by current methods used to kill adult laboratory rodents. Attention is given only to those methods used across scientifically advanced countries that have published and/or evidence-based guidance. Consideration will be given only to primary methods that have the potential to affect the animals’ conscious experience prior to loss of consciousness. In examining various methods available and in current use, this review will highlight welfare costs and benefits, comment on reliability and the challenges posed to operator health and safety. Finally, we describe emerging methodologies that may have potential for refinement and highlight knowledge gaps for prioritisation of future research efforts.

## Relevant definitions and terminology

The term ‘killing’ refers to any intentional act or process that results in the death of an animal.^12^ Euthanasia is considered to mean a ‘good death’ and therefore one without pain and suffering and, in some contexts, refers to a death that is in the animal’s interests (i.e. to end pain and suffering).^13–15^ The use of euthanasia throughout guidance and regulation protecting laboratory animals implies that approved killing methods are humane and may falsely alleviate potential public concern.^16^ As such, because there is considerable uncertainty about the welfare consequences of existing methods used to kill laboratory rodents, the universal term ‘killing’ will be used rather than ‘euthanasia’ throughout this review. A large component of the extent of suffering at killing depends upon the time to loss of consciousness.^17^ Loss of consciousness can be defined as the transition from a state of conscious awareness, where the animal is aware of their surroundings and is responsive to external stimuli, to unconsciousness, which occurs when the awareness of self and surroundings are lost, becoming unresponsive to external and internal stimuli.^17–19^ The majority of rodents are conscious during handling and the application of killing methods and are therefore capable of experiencing negative states (e.g. pain and fear) until they lose consciousness.^12,14,15^

## Assessing welfare at killing

Concern for animal welfare during killing stems from animals’ ability to experience potentially negative sensations occurring during the conscious phase of the process. To fully determine the welfare consequences of a killing method, it is important to determine (a) the time taken for the animal to be rendered unconsciousness inclusive of pre-handling time and kill method application and (b) the presence and character of potentially negative experiences during the conscious phase.

Behaviourally, it is possible to determine the transition from consciousness to unconsciousness by assessing the gradual loss of reflexes and goal-directed behaviours and ultimately, motor coordination. Generally, the cessation of movement and onset of recumbency is considered to be a marker of unconsciousness in animals, which is typically measured in rodents by their inability to effectively right their posture, referred to as loss of righting reflex or loss of posture.^20,21^ We can also measure changes in behavioural output, through the transition from goal directed behaviour (e.g. motivated escape behaviours)^22,23^ to the presence of spontaneous behaviours such as jumping, gasping etc., through to the presence of tonic and clonic convulsions in the unconscious phase.^24–26^ Neurophysiologically, we can measure brain activity to infer the likely degree of consciousness. Recordings of the electroencephalogram (EEG) provide a method for determining changes in global brain state,^27–29^ and appear to be common across vertebrates,^30,31^ where changes in signal amplitude and frequency reflect the transition between consciousness and unconsciousness.^32–35^ Unconsciousness is typically characterised by the presence of high amplitude, low frequency activity in the EEG signal (so-called slow wave activity), which, upon brain death, diminishes so that only low-level residual noise is present with no meaningful EEG signal (isoelectric).^34,36–38^

The presence of negative experiences can be assessed using both behavioural and physiological measures. Behaviourally, species-specific ethograms are employed, identifying behaviours associated with panic, fear, stress and/or anxiety, which have been extensively studied and validated.^3^ For example, the administration of an anxiolytic results in reduced frequency of fear behaviour (i.e. escape attempts).^39^ Physiological indicators include changes in the circulatory system and respiratory parameters, for example, increases are associated with the presence of negative emotions (e.g. stress and/or fear), and provide some indication of suffering at time of killing.^40,41^ However an unavoidable caveat to both behavioural and physiological measures is the difficulty in disentangling them from the dying process, given the obvious direct consequences of the method applied on both the cardiac and respiratory systems,^17,34,36^ as well as cognitive function.^42,43^ Therefore, although useful, the available welfare measures are not without their limitations, which must be considered carefully during study design and during the subsequent interpretation of their findings.

## Scientific, ethical and practical considerations for personnel

In addition to animal welfare consequences, each killing method is associated with important, scientific, ethical and practical implications, which the operator must carefully consider in evaluation, selection and application. Importantly, the method by which an animal is killed has the potential to affect scientific outcomes.^6,44^ For example, the administration of chemical compounds may have unwanted toxicological side effects for downstream molecular targets or unwanted pathological consequences in specific organs.^6,44^ Further, the emotional wellbeing of the operator must be considered. The action of killing an animal is potentially distressing,^45,46^ which, combined with the direct, non-aesthetic nature of some of the physical methods (e.g. cervical dislocation, decapitation, blunt force trauma) can have negative emotional consequences. This likely explains why some people are less comfortable with the application of physical methods compared with non-contact approaches.^47^ Furthermore, the safety and wellbeing of operators must be considered. Some methods pose greater risk to operator safety than others, for example anaesthetic agents that have specific health and safety implications.^48^ The success and welfare implications of some methods also relies heavily on the competence of staff, presenting serious risks with the quality of training and assessment. Thus, the goal of achieving an optimal killing method is multi-faceted, representing a balance between animal welfare outcomes, reliability, scientific integrity, operator safety and wellbeing of personnel.

## Cervical dislocation

Cervical dislocation involves the separation of the cervical vertebrae resulting in lethal trauma to the spinal cord. It is considered to induce rapid unconsciousness due to concussion and damage to the brain and/or cerebral ischemia.^49–52^ Typically, in rodents, it involves placing the finger (manual) or an instrument (mechanical) behind the base of the skull whilst pulling the tail firmly to achieve rapid separation of the high cervical vertebrae. As a physical method it is often considered aesthetically unpleasant,^47^ but it remains a common choice due to several advantages, including absence of toxicity associated with the administration of chemical compounds affecting scientific outcomes, rapid application and a lack of requirement for specialist equipment. However, there remains very little scientific evidence showing that cervical dislocation produces a reliable and/or a humane death.^53^ Assumptions that cervical dislocation offers a humane death arise from data extrapolated from the decapitation of rats,^54–58^ and data surrounding the specific welfare impacts of cervical dislocation remain sparse in rodents.^53,59^ This is particularly concerning given that cervical dislocation is very different from decapitation, with a different mode of action, and of course mice are not small rats. The few studies undertaken raise major concerns about the accuracy and efficacy of the dislocation and hence the success and reliability of the method.^53,59,60^ For mice, due to their small size, failure to apply dislocation accurately poses a serious risk, as reliably severing the cervical and not thoracic region of the spine can prove difficult.^53,59,60^ Indeed, studies have reported that 20–25% of mice exhibited thoracic rather than cervical fractures,^59,60^ and that 9.6% of examined mice exhibited no cervical dislocation at all.^59^ This is concerning because it is important to ensure high cervical spine dislocation to ensure rapid concussion, neurogenic shock, loss of consciousness and death.^53,61–63^ This was confirmed by Carbone et al.,^53^ who demonstrated that midthoracic dislocation alone did not induce respiratory arrest and death (100% failure rate). This is in line with previous work confirming inaccuracy of dislocation location when using three cervical targeting dislocation techniques (78% had thoracic and/or lumbar lesions), demonstrating an overall failure rate of 21%.^59,60^ There is, however, evidence to suggest that correctly performed cervical dislocation can induce rapid loss of consciousness and cortical function.^59^ Cartner, Barlow and Ness (2007) evaluated EEG amplitude and visually evoked potentials (VEPs) following cervical dislocation and decapitation in mice. Brain activity significantly decreased 5–10 s following cervical dislocation and 10–20 s following decapitation, leading to the conclusion that if cervical dislocation is done correctly, it is possible that it can result in quicker loss of cortical activity than decapitation.^53,59,64,65^ This is likely due to the concussive effect of cervical dislocation due to extensive widespread trauma to the spinal cord and brain stem evoking massive depolarisation of neurons and neurogenic shock.^61,62,65^

Work to date concurs that cervical dislocation is particularly susceptible to a high failure rate and that proper technique is crucial for an effective and high welfare method for killing laboratory rodents.^53,59,60^ Work from agriculture highlights the benefit of a tool to improve dislocation when dispatching poultry on farm.^66–68^ Therefore, more research is urgently needed to explore innovative method refinements to help standardise the technique, with the potential use of aids/tools to improve accuracy in order to ensure cervical rather than thoracic dislocation in laboratory rodents.

## Decapitation

Decapitation of conscious laboratory rodents has been controversial following findings reported by Mikeska and colleagues,^64^ who showed that brain activity was sustained for around 14 s after decapitation. Isoelectric activity, a marker commonly used to determine brain death, occurred after around 27 s. Furthermore, they suggested that the high frequency EEG signals observed were indicative of discomfort, pain and negative affective responses to decapitation. However, this claim remains highly controversial and unsubstantiated, and the assertion that such high frequency signals reflect pain and discomfort has been heavily criticised.^54,56,58^ One strong counterargument comes from findings showing that high frequency EEG signals are also present whilst under general anaesthesia,^33,69,70^ as well as during rapid eye movement (REM) sleep.^27^ Moreover, it has also been argued that, following decapitation, rapid blood loss would result in hypoxia rendering the decapitated head unconscious in less than 2.7 s,^55^ and that lack of blood supply would be unable to support ongoing brain activity.^64^ In subsequent EEG work, it took 17 s following decapitation for the EEG signal to become isoelectric, with the power of the frequency bands expressing cognitive activity (13–100 Hz) decreasing exponentially to less than 50% of baseline power, representative of an unconscious state, after 3.7 s.^58^ This result was corroborated in 1992 by loss of consciousness reported in 3–6 s,^56^ as well as disputed by a later study demonstrating that brain activity (EEG and VEP assessment) was sustained for relatively long periods following decapitation (15–20 s).^59^ EEG and VEP assessment are useful indicators but have limitations to their use. VEP assessment requires the application of a series of visual stimuli and as such provides a binary (yes or no) response to the presence of a visually evoked potential at designated discrete time points. Therefore, it does not provide a continuous response and does not necessarily correlate with the degree of consciousness as VEP signals in the visual cortex have been recorded despite being under desflurane anaesthesia.^17^ Further, for gradual killing methods, the transition between awake to unconsciousness represents a continuum. Therefore, the discrete nature of VEP makes recording the exact point of loss of consciousness implausible. At present it remains unclear which EEG patterns are representative of consciousness and not responsiveness (vigilance),^71^ and, therefore, their use for determining the welfare at time of killing are limited.^72^

Although decapitation is highly effective in terms of inducing a non-recovery state, controversy surrounding its ability to elicit pain and the duration that the brain remains conscious are still strongly debated and remain under investigation.^58,73^ Until conclusively proven, a conservative approach is to use upper durations for loss of consciousness to form conclusions about the techniques ability to induce a rapid and humane death. Currently, evidence suggests longer latencies to loss of consciousness than correctly performed cervical dislocation.

## Concussion of the brain by blunt force trauma

Concussion is a physical method that involves applying a severe blow to the skull with sufficient force to produce haemorrhage and depression of the central nervous system (CNS), rendering the animal unconscious instantaneously by concussion that disrupts normal brain function.^74,75^ Rapid acceleration of the head causes the brain to impact on the inside of the skull, disrupting electrical activity due to changes in intra-cranial pressure along with potential irreversible damage to blood vessels and nervous tissue.^76^ Like cervical dislocation, this method is highly reliant on the ability of the human operator to achieve a correctly targeted blow and is therefore susceptible to human error and potentially high failure rates.^77^ If the operator does not deliver sufficient force or strikes the incorrect anatomical location, then the strike is highly likely to induce pain and suffering if the animal remains conscious. It is important to highlight that this method is internationally considered a stun only, and must be immediately followed by another killing method such as cervical dislocation.^6,8,9^ This is because concussion is reversible, such that the animal could regain consciousness and recover.

Perhaps surprisingly, blunt force trauma has not been studied specifically for laboratory rodents, but some evidence can be extrapolated from other species. Most farm animals are stunned prior to exsanguination for slaughter, which is usually achieved by physical, electrical or gas approaches.^74,75^ Concussive stunning methods typically used for poultry provide the most meaningful comparison. In poultry, physical concussion is typically achieved by specialist hand held devices such as non-penetrating captive bolt guns which must be followed immediately by exsanguination or dislocation,^76^ and have been assessed extensively for animal welfare impacts using both physiological and behavioural responses.^30,78–80^ Turkeys stunned using three different concussive non-penetrating captive bolt guns showed a 94% success rate, with the turkeys rendered unconscious within 10 s, demonstrated by the significant reduction in total power of the EEG (<84%), followed shortly by isoelectric activity.^78^ However, two birds (6%) had EEG activity continuing for up to 60 s after the blow and demonstrated rhythmic respiration, neck tension and nictitating membrane reflexes, showing they were not concussed or killed immediately by these tools. Failure was proposed to be due to a combination of incorrect positioning of the instrument by the operator but also malfunction of the equipment itself, highlighting the importance of accuracy and correct functioning and maintenance of equipment. At present, there is no specialist equipment commercially available to deliver a consistent concussive blow for use in laboratory rodents, although a single recent study assessed the performance of a penetrating bolt gun in guinea pigs.^81^ Blunt force trauma therefore requires scientific validation in laboratory rodents to confirm the specific techniques and forces required to induce immediate unconsciousness, to safely underpin their continued use in a laboratory setting. It is crucial that future work focuses on investigating failure rates, operator variability and species differences in relation to bodyweight and anatomy, in addition to methodological factors such as determining the actual force applied by existing techniques. Furthermore, it may be beneficial to investigate whether the use of specialist tools could be adapted for use in rodents and offer advantages for achieving a consistent and effective concussive blow. It is possible that, if done correctly, with sufficient and consistent force, using specialist tools to mitigate against operator variability, then concussion could provide a high welfare method of killing, as outlined in previous work in poultry.^82,83^

## Exposure to CO_2_ gas in a rising concentration

Exposure to a rising concentration of CO_2_ is the most commonly used technique for killing laboratory rodents.^13,84^ Some systems are fully automated and enable the animals to be killed in their home cage along with their cage mates, which offers several advantages over physical methods, such as its high-throughput and non-contact nature, elimination of stress associated with handling, isolation and restraint, as well as minimising the impact of operator error.^85–87^

Inhalation of CO_2_ has wide-ranging effects on the respiratory, circulatory and nervous systems. At low concentrations (5–35%) it causes hyperventilation, bradycardia and hypertension and results in increased activity of the hypothalamic-pituitary-adrenal (HPA) axis via activation of glucocorticoid receptors.^86,88–90^ At higher concentrations, hyperventilation is followed by depression and failure of the circulatory and respiratory systems, resulting in an anaesthetised state before death due to neuronal acidification, reduced intracellular pH and hypoxia.^86^ Although used commonly in laboratory and agricultural contexts, killing animals by exposing them to CO_2_ is a source of growing welfare concern.^13,72,77,84^ Specific potential welfare insults arise from the capacity of CO_2_ to induce negative sensations and experiences such as pain, fear, anxiety and respiratory distress, breathlessness (dyspnoea) and air hunger, all of which have been reviewed elsewhere and will be expanded upon here.^13,72,84,91,92^ The discussion below will focus on a brief summation of the literature with regards to pain, negative affective states such as fear and anxiety and consideration with regards to severity versus duration for methodological factors such as fill method and flow rate.

### Evidence of pain

The suggestion that exposure to CO_2_ is painful for animals arose initially from work conducted on humans that reported concentrations of around 50% CO_2_ as painful and capable of inducing distress.^88,93,94^ Subjects judged increasing concentrations of CO_2_ progressively more noxious, from ‘highly unpleasant’ at 50% to ‘painful’ at 100% CO_2_.^93^ The pain associated with exposure to CO_2_ is likely due to the formation of carbonic acid when gaseous CO_2_ comes into contact with moist tissues and mucous membranes, specifically within the nasal and ocular epithelia.^93–96^ These concerns are easily extrapolated to animals exposed to CO_2_ via identical mechanisms.^93,95,96^

Rodents have similar nociceptors in the mucous membranes and at comparable densities that respond to CO_2_ at similar concentrations as in humans.^93–97^ Therefore, it seems reasonable to assume that concentrations above 50% are also painful for laboratory rodents and, according to Leach,^99^ in humans and rats, most nociceptors are activated at a concentration of 40% CO_2_. In addition, pain researchers have for many years used exposure to CO_2_ to induce pain in laboratory rodents and therefore it is an accepted noxious stimulus at concentrations above 25%.^95,96^ However, the rate of exposure can determine whether the animal loses consciousness prior to activation of nociceptive activity, making it a crucial factor in evaluating the welfare impact of this methodology.

In laboratory rodents, there is evidence that exposure to CO_2_ induces rapid loss of consciousness at concentrations above 40% and cessation of life occurs above 70%.^99^ This is why guidelines recommend that animals are exposed to a rising concentration of CO_2_ (i.e. 20% of the chamber volume per minute) rather than exposed to a pre-filled chamber in order to mitigate against exposure to high CO_2_ concentrations prior to loss of consciousness.^6,8,9^

Although most of the evidence demonstrating that CO_2_ exposure induces pain is associated with exposure to high concentrations, some evidence has suggested that low levels of CO_2_ are also capable of inducing pain. Increased neural firing in the medullary dorsal horn (a pain-sensing area) has been demonstrated upon exposure to 25% CO_2_, with activity increasing linearly with increasing CO_2_.^95^ Therefore, there is potentially overlap between anaesthetic and nociceptive concentrations such that pain could be experienced before loss of consciousness during gradual fill application.

### Evidence of fear and/or anxiety

When an animal is exposed to a stressful situation, a series of well-understood signalling events are activated, preparing the body for a ‘fight or flight’ response. Acute stress results in activation of both the sympathetic-adrenal-medullary system (SAM) and the HPA axis, and the release of a number of neurotransmitters and hormones (e.g. norepinephrine and corticosterone).^100^ This results in autonomic physiological responses, including increased heart rate and blood pressure, in addition to measurable changes in the hormones and neurotransmitters themselves. Exposure to CO_2_ induces a stress response as indicated by initial increases in heart rate and blood pressure,^89,101^ before bradycardia is observed as the anaesthetic properties of CO_2_ occur.^86^ Changes in tissue histology, decreased blood pH indicative of acidosis and increases in plasma corticosterone are also observed,^93,102–104^ indicative of a possible stress response. Although these measures are useful, they cannot necessarily indicate the animal’s subjective experience and are difficult to interpret for two primary reasons. First, these responses lack emotional valence, whereby both positive and negative events can result in the same physiological outcome, e.g. increased heart rate in both exciting and stressful situations.^105^ Second, it is often difficult to disentangle the physiological consequences of stress from the killing process because dying has obvious consequences for several physiological systems, such as depression of respiratory and cardiac responses and activation of key reflexes.

To circumvent these issues, a large number of studies have measured changes in behaviour upon exposure to CO_2_.^22,25,98,99,106–110^ Spontaneous behaviour provides important information with regards to how exposure to CO_2_ affects normal behavioural repertoire, and the presence of concerning behaviours (e.g. escape attempts) can inform us of the animal’s likely experience. Spontaneous behaviours such as increased locomotion, jumping, rearing, gasping, defecation and urination, escape behaviours and seizures have all been reported when animals are exposed to CO_2_.^37,98,99,106^ However, although informative, it remains difficult to infer the animals experience objectively, and, crucially, it cannot be easily determined whether certain behaviours are elicited during the conscious or unconscious phase of CO_2_ exposure.

Application of behavioural paradigms that are focussed on goal directed (active) rather than spontaneous (passive) behaviours may provide a route to better understand the experience of rodents when exposed to CO_2_. These include approach-avoidance and aversion-avoidance paradigms. They differ from one another in terms of their motivations for either a food reward (approach-avoidance) or their motivation to avoid a known aversive stimulus such as bright light (aversion-avoidance), and both aim to provide a way of measuring the degree of aversion expressed by an animal when exposed to a situation.^22,25,98,108–114^

The approach-avoidance paradigm has been used widely and involves the animal choosing to remain in a chamber in order to obtain a food reward or choosing to forego the reward in order to leave the chamber.^25,98,99,109,112–114^ By contrast, aversion avoidance testing involves the animal choosing to leave the environment in order to access a separate aversive environment, such as exposure to a brightly lit compartment.^110,111,113^ The premise of these tests is that they induce a motivational conflict, and therefore the stronger the motivation to escape the greater the aversion is deemed to be.^112^ The majority of studies employing these paradigms have shown that rodents will actively avoid exposure to CO_2_,^98,99^ even if this means spending time in an environment they find aversive,^22,110,111^ or foregoing a food reward whilst under food-deprived conditions.^22,25,108–110,112,114^ In approach-avoidance tests, the strength of aversion can only be measured if the incentive of the reward is known, and therefore use of an appropriate reward is crucial to the interpretation of findings.^112,114^ Another important consideration is the strength of the animals’ feeding motivation and the fact that this can be manipulated by the experimenter makes it an attractive choice. It is hypothesised that if severely food-deprived rats are not willing to tolerate the stimulus (CO_2_ exposure), then their aversion to the stimulus must be strong. However, Kirkden et al. highlighted that the relationship between food deprivation and motivation may be more complex.^112^ They found that the animal’s willingness to remain in the chamber filling with CO_2_ did not increase with increasing food deprivation, and past a certain deprivation level (7–7.5 h), the animals motivation to remain was found to decrease despite increasing hunger.^112^ Thus, although this paradigm still proves a useful tool to determine an animal’s aversion to a given stimulus, caution must be applied when making inferences about the strength of that aversion. Aversion-avoidance testing is not without its limitations in this regard; the latency to exit the chamber can be influenced by a number of factors, including onset of ataxia due to the anaesthetic effect of CO_2_.^115^ Although these paradigms potentially provide important information with regards to an animal’s motivation to avoid CO_2_, careful interpretation and design of studies are crucial to their use.

A major factor considered to cause distress and feelings of panic and/or fear upon exposure to CO_2_ is the sensation of breathlessness, referred to as dyspnoea, with the most debilitating component of dyspnoea referred to as ‘air hunger’.^88,91,116,117^ Dyspnoea is known to be an unpleasant sensation and highly distressing in humans, where people have reported the sensation of not being able to get a full breath when exposed to concentrations of CO_2_ above 8%.^88,116^ Air hunger describes the conscious appreciation of an urge to breathe and, in humans, is associated with anxiety, frustration and fear.^117^ Therefore, it is plausible that air breathing mammals too can experience feelings of dyspnoea, raising a significant welfare concern during CO_2_ exposure, even at relatively low concentrations.^91^ However, there is a lack of research on the presence and magnitude of this phenomenon in non-human animals and is an extremely difficult phenomenon to measure.^116,117^

### Severity versus duration: prefill versus gradual exposure

Current guidelines (EU, UK, USA, Australia and New Zealand) recommend that rodents must be gradually exposed to CO_2_ in a rising concentration rather than placed in chambers that have been prefilled with the gas.^6,8,9,118–120^ Gradual fill is intended to result in a slowly increasing concentration of CO_2_ until the animal loses consciousness, to mitigate against the exposure to high concentrations associated with pain,^6,8,9^ but notably this approach would not prevent dyspnoea and air hunger.

When considering the term ‘gradual exposure’, there is considerable ambiguity in the classification of what constitutes ‘gradual’. Several studies have focussed on determining what flow rates might offer the best approach when killing laboratory rodents using CO_2_. Flow rate affects the speed at which an animal loses consciousness (faster flow rates mean that animals often lose consciousness quicker than slower flow rates),^121^ and the duration of the period where suffering is a possibility. This effect is thought to be mediated by the rate of change in pH in the cerebrospinal fluid that underlies loss of consciousness.^25^ Therefore, if concern for negative sensations such as dyspnoea and air hunger were not upheld to limit suffering due to pain, a balance must be struck between the time to loss of consciousness and the concentration of CO_2_ at which this occurs.

Whether or not employing a gradual fill does in fact avoid fear, anxiety and pain in rodents has been the focus of several studies that have employed interpretation of spontaneous behaviour and preference testing.^25,26,87,89,101,114,122–124^ Findings have been contradictory, with some studies reporting no signs of behavioural distress upon gradual CO_2_ exposure,^87,89,122^ while others have reported signs of distress and/or dyspnoea.^25,26,41,101,123,124^ Contradictory findings are likely mediated by different methodological factors such as fill location (top versus bottom fill), in addition to different definitions of behavioural distress. A variety of flow rates have also been investigated. Niel et al. investigated flow rates ranging between 3% to 27% of the chamber volume per minute and found that rats left the chamber at all flow rates, and no rat remained in the chamber until loss of consciousness.^25^ This indicates that even the most gradual flow rate investigated (3%) was aversive, which is at odds with current recommendations that exceed such exposure.^6–9^

A significant body of evidence supports the conclusion that exposure to CO_2_ is aversive for laboratory rodents. Whether slower or faster flow rates of CO_2_ are more aversive remains controversial and therefore the suitability of current guideline recommendations is unclear. Focus should be on the trade-off between duration and severity; specifically, whether longer durations with lower severity are optimal compared with shorter durations with higher severity. However, the ability to quantify an animal’s degree of aversion remains difficult. An obvious and unavoidable consequence of exposing animals to CO_2_, is that the animal succumbs to the agents’ chemical properties and quickly loses its ability to show its level of aversion. At this time, it is possible that the animal remains fully conscious, experiencing negative sensations but unable to respond accordingly. Given the limited scope for refinement with CO_2_, future research should focus on developing alternative high-throughput and non-contact methodologies capable of providing laboratory rodents with a higher welfare death. One methodology that may offer promise is hypobaric hypoxia achieved by gradual decompression. A recent study evaluating the pathological and behavioural consequences of gradual decompression in anaesthetised laboratory mice demonstrated the methods ability to elicit a non-recovery state (100% kill success) with minimal pathological consequences.^125^ However, given that the animals remained anaesthetised during exposure, the full welfare consequences remain to be elucidated.

## Exposure to CO

Fatal hypoxia is induced by CO, as it binds irreversibly to iron in haemoglobin, blocking the uptake of oxygen by erythrocytes.^85^ CO is colourless, tasteless and lacks odour, so insidious exposure is highly dangerous and a serious risk to human health and safety. Little research has focussed on whether the exposure of laboratory rodents to CO provides a humane methodology for killing purposes and research from other species including pigs, cats and dogs reported agitation during the conscious phase.^126–128^ Only one study has determined the aversion to CO induced hypoxia in laboratory rats at various flow rates (3–7%).^23^ The study found that most rats chose to avoid CO; however, 1 rat out of 21 remained in the test cage until recumbency at the highest flow rate and 1 rat became recumbent immediately after exiting the chamber following exposure to a 6% flow rate. In a follow-up study, latency to recumbency for the same three flow rates (without the possibility for escape) was investigated, demonstrating that latency was shorter when exposed to CO compared with CO_2_ and inhalant anaesthetic gases such as isoflurane.^84,89,129^ However, all rats exposed to CO showed behavioural signs of aversion and all rats exhibited convulsions after recumbency. Whether the animals were unconscious whilst recumbent was not determined given the lack of neurophysiological data; however, convulsions usually occur after loss of consciousness.^23^ Given the lack of evidence supporting its benefit over CO_2_ for rodent welfare, combined with its high risk to human health and safety, CO remains a method that is rightly avoided on safety grounds.

## Overdose of an anaesthetic

Anaesthetic agents used for killing laboratory rodents fall into two classes: barbiturates or halogenated anaesthetic agents. Barbiturates, most commonly sodium pentobarbital, are injectable agents and are typically administered at large doses via either intraperitoneal or intravenous routes.^6,8^ By comparison, halogenated anaesthetic agents such as halothane, enflurane, isoflurane and sevoflurane are all inhalational agents and, when administered in high concentrations in oxygen, lead to overdose and death. The welfare concerns associated with anaesthetic agents relate to the chemical properties of the compounds themselves and the requirement for large doses and thus their ability to induce pain and discomfort.^6,130,131^

The most common anaesthetic agents used to kill laboratory rodents are barbiturates, typically, sodium pentobarbital administered intraperitoneally.^130^ Its mechanism of action involves depressing the central nervous system by acting on GABA_A_ receptors, ultimately leading to loss of consciousness as assessed by loss of the righting reflex within approximately 104–140 s,^131^ and respiratory and cardiovascular depression and cessation within approximately 283–485 s depending upon the dose.^130–135^ Pentobarbital is highly alkaline (pH ∼10), leading to the suggestion that its administration into the peritoneal cavity is likely to be associated with discomfort and pain.^136^ Indeed, increased neuronal expression of c-fos-like immunoreactivity in the spinal dorsal horn has been demonstrated with intraperitoneal injection of sodium pentobarbital, suggesting greater nociceptive activation compared with rats that also received a local anaesthetic.^136^ As such, administration of local anaesthetics may offer some amelioration of irritation and pain. Lidocaine or bupivacaine decreased abdominal writhing in rats without affecting the latency to induce unconsciousness and without causing unwanted effects on scientific outcomes such as in immunohistochemical assays.^130,136^

In the only study to date focussing on assessing the behaviours of laboratory mice post injection with sodium pentobarbital, Dutton et al. concluded that laboratory mice showed no behavioural signs of pain when sodium pentobarbital was injected subcutaneously into the hind paw.^137^ However, this study focused only on specific behaviours considered to be indicative of pain in laboratory mice (i.e. abdominal writhing), rather than a full behavioural ethogram following injection, and it is possible that some pain-related behaviours were not identified. A full behavioural assessment should be conducted alongside control animals that are administered analgesics/local anaesthetics before making welfare inferences.

An additional concern relates to the potential experience of distress and discomfort by the animal due to a combination and accumulation of procedures: handling, restraint and needle puncture. For laboratory mice, restraint is usually achieved by scruffing, whereas for rats, two people are usually required, with the rat restrained using two hands (one over the shoulders and one holding the rear legs) and a second operator performing the injection, both of which have been evidenced as stressful.^138–140^ Therefore, when assessing the welfare impact of injectable agents, researchers should incorporate the stress associated with handling and restraint of the animal.

Inhalational anaesthetic agents are non-contact and so mitigate against some of these issues; however, there are concerns related to their possible aversive properties. A number of studies have focussed on whether various anaesthetic gases (e.g. enflurane, halothane, sevoflurane, etc.) offer a humane death, especially compared with exposure to CO_2_.^13,85,98,99,109,111,112,121,129,141^ However, findings from various studies are not in agreement; with some suggesting that these inhalational agents offer a possible refinement over CO_2_, with animals losing consciousness rather than foregoing a food reward,^129^ or escaping to a brightly lit aversive compartment,^113,129^ whereas others have demonstrated that, as with CO_2_, rodents will actively avoid a chamber filling with halogenated anaesthetic agents.^98,99,109,113,121,129,141^ Compared with CO_2_, studies have shown that mice exposed to isoflurane and sevoflurane exhibited greater vocalisations, stress induced grooming and activation of neuroendocrine responses resulting in elevated adrenaline, noradrenaline, ACTH and corticosterone plasma concentrations.^121,141^ Furthermore, the degree of aversion has been found to increase upon repeated exposure,^111,112,129,142^ whereby both rats and mice will actively avoid the agent if already exposed to it once before, which is of particular concern given the likelihood of pre-exposure through general anaesthesia for scientific procedures.

It is possible that rats may benefit from the administration of local anaesthetics alongside sodium pentobarbital, although further investigation is needed for laboratory mice. Evidence demonstrating that the use of inhalational anaesthetic agents offers a possible refinement over CO_2_ remain controversial. This is likely due to differences in aversion testing protocols and/or potential differences in anaesthetic and oxygen flow rates. Future research must explore different flow rates to assess whether slower rates of induction result in lower levels of aversion. Although some evidence suggests this is possible, greater aversion upon re-exposure remains a significant concern for welfare.

## Microwave irradiation

Focused beam microwave irradiation used for killing purposes is a relatively new method and is permitted for laboratory rodents only in the US.^143^ It involves rapidly and remotely heating the brain by application of a high energy beam, halting brain enzyme activity and inducing loss of consciousness due to diathermal syncope.^6,144,145^ The approach was first used in neurobiology studies for fixing the brain and its metabolites, whilst maintaining anatomic integrity of the tissue;^146–148^ however, it has recently gained traction for the use in the commercial stunning of cattle.^144^ Presently, one of the greatest limitations to its use is the high cost of the equipment. Although welfare has not been directly assessed when using this methodology, it has been suggested that the animal loses consciousness and is killed rapidly (approximately 600–900 ms for rats and 100–330 ms for mice.^146^ However, the full welfare consequences of the technique are yet to be fully explored, including factors such as pre-handling and restraint. Further research should focus on determining the latency to loss of consciousness, along with other negative welfare consequences as well as operational risks (e.g. failure rate).

## Conclusions

This review critically evaluates the welfare outcomes associated with currently available methodologies used to kill laboratory rodents. The range of considerations involved demonstrate that careful consideration of a range of factors is important when selecting an appropriate methodology that protects both animal welfare and scientific integrity. The available evidence suggests that researchers and laboratory personnel should seek to avoid killing laboratory rodents by exposing them to CO_2_, given the plentiful evidence of aversion, even at low concentrations and flow rates. Substantial questions surrounding this technique’s ability to provide a humane death persist, calling into question its approved status and extensive use worldwide. Future work should be focussed on the development of a humane high-throughput alternative. Until then, the use of CO_2_ may be warranted only as a high-throughput methodology, especially if the alternative was to employ physical methods, which rely on the success of human operators and may not achieve reliable successive kills. One methodology that may offer promise is hypobaric hypoxia achieved by gradual decompression;^125^ however, further work is needed in conscious animals to elucidate the welfare consequences before recommendation and consideration in legislation can commence. At present, policies, guidelines and/or legislation do not include classification of circumstances. Until an alternative is fully validated, we suggest policymakers and stakeholders should advocate for the removal of CO_2_ from existing permitted methods of humane euthanasia, unless institutions provide additional rationale for its use (e.g. exploiting its high-throughput nature).

Although the evidence base surrounding physical methods is somewhat lacking in comparison with CO_2_, existing work does encourage the possibility that some of these provide a fast death with minimal negative experiences. However, successful cervical dislocation, blunt force trauma or decapitation rely on the technique being performed correctly by the operator. A major limitation with physical methods especially is the accuracy of the technique, leading to a high error rate in their application. This supports the need for standardised and independently quality-controlled training programmes to facilitate optimal success rates, in addition to new research focussed on developing aids and/or specialist tools to help improve their uptake, accuracy and success rate. The evidence presented in this review leads to questions around the inconsistent inclusion criteria and acceptability of cervical dislocation, blunt force trauma and decapitation in regulation and/or guidance. Perhaps the reasons are purely humancentric, since decapitation poses more risk to operator health and safety via direct injury and exposure to pathogens. It is also an emotive and understood term by the general public. These issues probably represent a case where potential benefits to animal welfare are outweighed by the risks to human safety and poor public acceptance.

More generally, research is also urgently needed to allow improved assessment of time to loss of consciousness (including the development of novel methods to accurately assess this) in laboratory rodents if we are to bring about meaningful changes to existing guidelines and policies. Furthermore, methodologies demonstrating negative affective states should be validated using species-specific ethograms and appropriate analgesic controls, as well as recognising method-specific differences in responses (e.g. recumbency ≠ unconscious).

Finally, there needs to be better outreach and dissemination of research findings, specifically with regards to advances focussed on making refinements to existing methods. Better review of new data and championing of open access scientific resources is crucial if we are to advocate best practice and have this reflected in policy and legislation. It is evident that there is lack of cohesion across countries and lack of a comprehensive ‘gold standard’ methodology. The inclusion of conditionally acceptable methods in guidelines across the US, Canada, Australia and New Zealand presents significant concern for animal welfare given their increased potential of providing an inhumane death if performed inadequately. We recommend that industry organisations, stakeholders and governments take a collaborative approach to evaluate and disseminate refinements to ensure all laboratory rodents are killed in the most humane manner currently available (Table 2).

**Table 2.** Summary for commonly used killing methods for laboratory rodents according to physical or chemical properties. Rapidity: ++ very fast, + fast, − slow; Efficacy: ++ very effective, + effective, − not effective.

Killing method	Species and limits	Mechanism of death	Rapidity/suggested time to loss of consciousness	Efficacy/kill success	Disadvantages	Other considerations
Cervical dislocation	Rodents up to 500 g (UK) or 1 kg (EU); however, prior use of a sedative required for rodents >150 g.Preference of sedative issued in US and Canada, not permitted for rodents over 200 g.	Physical separation of the brain and CNS resulting depression of the CNS and damage to the brain stem	++	+	Large operator error leading to high failure rate. Aesthetically unpleasant	Adequate training is essential
Decapitation	All rodents	Physical disruption of the brain from the CNS; followed by anoxia due to rapid blood loss	++	++	Potential for operator error and safety concernsAesthetically unpleasant	Adequate training and maintenance of equipment essential.Primarily used when pharmacological agents and CO_2_ are contraindicated.
Concussion by blunt force trauma	Rodents up to a weight of 1 kg	Used to render the animal unconscious then must be followed by another method.Haemorrhage and depression of the CNS.	++	+	Aesthetically unpleasantNo direct evidence-base to support the use of this method in laboratory rodents; assumed to be a humane method due to translation from farm animals	Adequate training is essentialMust be followed by another killing method
Exposure to CO_2_ gas	Rodents up to 1.5 kg (but not neonatal rodents)	Respiratory acidosis, decreased intracellular pH followed by cardiac arrest.	+	++	Stress/distress and pain associated with exposure to CO_2_	Advantageous as provides a high-throughput method with the option to kill animals in their home cage environmentFill rate and other procedural factors must be considered as differ across EU, US, and Canada
Exposure to CO gas	All rodents	Fatal hypoxia due to inability for effective gas exchange. CO binds to haemoglobin in erythrocytes preventing effective binding of oxygen.	+	++	Evidence of agitation and convulsions across multiple species including laboratory rodents. Unclear whether these occur during conscious period. Serious concerns for personnel safety due to toxicity and explosive nature of the gas.	Advantageous as provides a high-throughput method with the option to kill animals in their home cage environment
Overdose of anaesthetic (via inhalant and injectable routes)	All rodents	Dependent upon chemical agent	+	++	Stress/distress associated with handling and administration of the compoundIrritation/pain dependent upon the agent administered.	Adequate training for correct restraint and injection route
Microwave irradiation	All rodents	Ultra-rapid destruction of the brain and brain enzymes by rapid heating	++	?	Requires specialist expensive equipmentFull assessment of ability to provide a humane death yet to be determined.Dangerous for the operator	Requires specialist trainingCommonly used when fixation of brain metabolites are required without losing integrity of the brain

CNS, central nervous system; CO, carbon monoxide, CO_2_, carbon dioxide; EU, European Union; US, United States.

## Data Availability

Data sharing is not applicable to this article as no new data were created or analysed in this study.

## References

[1] Knight A. Non-animal methodologies within biomedical research and toxicity testing. ALTEX 2008; 25: 213–231. 18841317 10.14573/altex.2008.3.213

[2] Cronin M. Non-animal approaches – the way forward. *Report on a European Commission Scientific Conference, 6–7 December 2016, Brussels,* 2019, https://data.europa.eu/doi/10.2779/373944

[3] Bryda EC. The mighty mouse: the impact of rodents on advances in biomedical research. Mo Med 2013; 110: 207–211. 23829104 PMC3987984

[4] Taylor K Alvarez LR. An estimate of the number of animals used for scientific purposes worldwide in 2015. Altern Lab Anim 2019; 47: 196–213. 32090616 10.1177/0261192919899853

[5] European Commission. Summary Report on the statistics on the use of animals for scientific purposes in the Member States of the European Union and Norway in 2018. Brussels; European Commission, 2021.

[6] AVMA. AVMA guidelines for the euthanasia of animals: 2020 edition. Members of the Panel on Euthanasia AVMA Staff Consultants. Schaumburg, IL: AMVA, 2020.

[7] ANZCCART. Euthanasia of animals used for scientific purposes Australian and New Zealand Council for the Care of Animals in Research and Teaching. Adelaide: ANZCCART, 2001.

[8] CCAC. CCAC guidelines on: euthanasia of animals used in science. Canadian Council on Animal Care in science. Ottowa: CACC, 2010.

[9] Home Office. Animals (Scientific Procedures) Act 1986. London: Home Office, 1986.

[10] Višak T Garner R. The ethics of killing animals. USA: Oxford University Press; 2016. 252 p.

[11] European Commission. Euthanasia of Experimental Animals. Luxembourg: Office for Official Publications of the European Communities, 1997.

[12] EC. COUNCIL REGULATION (EC) No 1099/2009 of 24 September 2009 on the protection of animals at the time of killing. Off J Eur Union 2009; 1–30.

[13] Hawkins P Prescott MJ Carbone L , et al. A good death? Report of the second Newcastle meeting on laboratory animal euthanasia. Animals (Basel) 2016; 6: 50. 27563926 10.3390/ani6090050 PMC5035945

[14] Leary S Underwood W Anthony R , et al. AVMA Guidelines for the Euthanasia of Animals: 2020 Edition. 2020. pp. 82–91.

[15] Leary S Underwood W Anthony R , et al. AVMA guidelines on euthanasia. Am Vet Med Assoc 2007; 218: 1–39.

[16] Sivula CP Suckow MA. Euthanasia. In: Weichbrod RH, Thompson GA(H), Norton JN (eds) *Management of animal care and use programs in research, education, and testing*. 2nd ed. Boca Raton (FL): CRC Press/Taylor & Francis, 2018, pp. 827–840. 29787045

[17] Meyer RE. Physiologic measures of animal stress during transitional states of consciousness. Animals (Basel) 2015; 5: 702–716. 26479382 10.3390/ani5030380 PMC4598702

[18] Boly M Seth AK Wilke M , et al. Consciousness in humans and non-human animals: recent advances and future directions. Front Psychol 2013; 4: 625. 24198791 10.3389/fpsyg.2013.00625 PMC3814086

[19] Pennartz CMA Farisco M Evers K. Indicators and criteria of consciousness in animals and intelligent machines: an inside-out approach. Front Syst Neurosci 2019; 13: 25. 31379521 10.3389/fnsys.2019.00025 PMC6660257

[20] McCarren HS Moore JT Kelz MB. Assessing changes in volatile general anesthetic sensitivity of mice after local or systemic pharmacological intervention. J Vis Exp 2013; 2013: 51079. 10.3791/51079 PMC3940706 24192721

[21] Nagasaka Y Wepler M Thoonen R , et al. Sensitivity to Sevoflurane anesthesia is decreased in mice with a congenital deletion of guanylyl cyclase-1 alpha. BMC Anesthesiol 2017 14; 17: 76. 10.1186/s12871-017-0368-5 PMC5471676 28615047

[22] Makowska IJ Weary DM. Using rat behavior to assess aversion to euthanasia agents. ALTEX 2012;(Proceedings of WC8): 465–467.

[23] Makowska IJ Weary DM. Rat aversion to carbon monoxide. Appl Anim Behav Sci 2009; 121: 148–151.

[24] Moody CM Makowska IJ Weary DM. Testing three measures of mouse insensibility following induction with isoflurane or carbon dioxide gas for a more humane euthanasia. Appl Anim Behav Sci 2015; 163:183–187.

[25] Niel L Stewart SA Weary DM. Effect of flow rate on aversion to gradual-fill carbon dioxide exposure in rats. Appl Anim Behav Sci 2008; 109: 77–84.

[26] Niel L Weary DM. Behavioural responses of rats to gradual-fill carbon dioxide euthanasia and reduced oxygen concentrations. Appl Anim Behav Sci 2006; 100: 295–308.

[27] Jing W Wang Y Fang G , et al. EEG bands of wakeful rest, slow-wave and rapid-eye-movement sleep at different brain areas in rats. Front Comput Neurosci 2016; 10: 79. 27536231 10.3389/fncom.2016.00079 PMC4971061

[28] Rensing N Moy B Friedman JL , et al. Longitudinal analysis of developmental changes in electroencephalography patterns and sleep-wake states of the neonatal mouse. PLoS One 2018; 13: e0207031. 30399187 10.1371/journal.pone.0207031 PMC6219806

[29] Aydin-Abidin S Yildirim M Abidin I , et al. Comparison of focally induced epileptiform activities in C57BL/6 and BALB/c mice by using in vivo EEG recording. Neurosci Lett 2011; 504: 165–169. 21964381 10.1016/j.neulet.2011.09.030

[30] Verhoeven MTW Gerritzen MA Hellebrekers LJ , et al. Indicators used in livestock to assess unconsciousness after stunning: a review. Animal 2014; 9: 320–330. 25354537 10.1017/S1751731114002596 PMC4299535

[31] Pierre LN Emilie B Boissy A , et al. Animal consciousness. Anim Welf 2018; 27: 87.

[32] Voss L Sleigh J. Monitoring consciousness: the current status of EEG-based depth of anaesthesia monitors. Best Pract Res Clin Anaesthesiol 2007; 21: 313–325. 17900011 10.1016/j.bpa.2007.04.003

[33] MacIver MB Bland BH. Chaos analysis of EEG during isoflurane-induced loss of righting in rats. Front Syst Neurosci 2014; 8: 203. 25360091 10.3389/fnsys.2014.00203 PMC4199270

[34] Martin JE Christensen K Vizzier-Thaxton Y , et al. Behavioural, brain and cardiac responses to hypobaric hypoxia in broiler chickens. Physiol Behav 2016; 163: 25–36. 27117817 10.1016/j.physbeh.2016.04.038

[35] Franken P Malafosse A Tafti M. Genetic variation in EEG activity during sleep in inbred mice. Am J Physiol 1998; 275: R1127– R1137. 9756543 10.1152/ajpregu.1998.275.4.R1127

[36] McKeegan DEF Reimert HGM Hindle VA , et al. Physiological and behavioral responses of poultry exposed to gas-filled high expansion foam. Poult Sci 2013; 92: 1145–1154. 23571322 10.3382/ps.2012-02587

[37] Gent TC Detotto C Vyssotski AL , et al. Epileptiform activity during inert gas euthanasia of mice. PLoS One 2018; 13: e0195872. 29672545 10.1371/journal.pone.0195872 PMC5908136

[38] Detotto C Isler S Wehrle M , et al. Nitrogen gas produces less behavioural and neurophysiological excitation than carbon dioxide in mice undergoing euthanasia. PLoS One 2019; 14: 1–15. 10.1371/journal.pone.0210818 PMC6354991 30703117

[39] Spiacci A Vilela-Costa HH Sant’Ana AB , et al. Panic-like escape response elicited in mice by exposure to CO_2_, but not hypoxia. Prog Neuropsychopharmacol Biol Psychiatry 2018; 81: 178–186. 29111406 10.1016/j.pnpbp.2017.10.018

[40] Nichols KE Holliday-White KL Bogie HM , et al. Cardiovascular and metabolic responses to carbon dioxide euthanasia in conscious and anesthetized rats. J Am Assoc Lab Anim Sci 2020; 59: 742–749. 32873368 10.30802/AALAS-JAALAS-19-000166 PMC7604681

[41] Powell K Ethun K Taylor DK. The effect of light level, CO_2_ flow rate, and anesthesia on the stress response of mice during CO_2_ euthanasia. Lab Anim 2016; 45(10):386–395. 10.1038/laban.1117 27654690

[42] Fenwick DC Blackshaw JK. Carbon dioxide as a short-term restraint anaesthetic in rats with subclinical respiratory disease. Lab Anim 1989; 23: 220–228. 2503658 10.1258/002367789780810590

[43] Kohler I Meier R Busato A , et al. Is carbon dioxide (CO_2_) a useful short acting anaesthetic for small laboratory animals? Lab Anim 1999; 33: 155–161. 10780819 10.1258/002367799780578390

[44] Canadian Council on Animal Care. Additional information on effects of euthanasia methods on research results. Addendum to the CCAC guidelines on: euthanasia of animals used in science, https://norecopa.no/3r-guide/ccac-guidelines-on-euthanasia-of-animals-used-in-science (2010, accessed 02 July, 2020).

[45] Holmberg T. Mortal love: care practices in animal experimentation. Fem Theory 2011; 12: 147–163.

[46] Herzog H. Ethical aspects of relationships between humans and research animals. ILAR J 2002; 43: 27–32. 11752728 10.1093/ilar.43.1.27

[47] Hickman DL Johnson SW. Evaluation of the aesthetics of physical methods of euthanasia of anesthetized rats. J Am Assoc Lab Anim Sci 2011; 50: 695–701. 22330717 PMC3189674

[48] Dehghani F Kamalinia M Omidi F , et al. Probabilistic health risk assessment of occupational exposure to isoflurane and sevoflurane in the operating room. Ecotoxicol Environ Saf 2021; 207: 111270. 32949927 10.1016/j.ecoenv.2020.111270

[49] Linares JA Dougherty S Millman S. Poultry welfare assessment on the farm: focusing on the individual. In: Mensch JA (ed) *Advances in poultry welfare*. Amsterdam: Elsevier; 2018, pp. 131–148.

[50] Gagea-Iurascu M Craig S. Euthanasia and necropsy. In: Suckow MA, Stevens KA, Wilson RP (eds) *The laboratory rabbit, guinea pig, hamster, and other rodents.* Amsterdam: Elsevier, 2012, pp. 117–139.

[51] Erasmus MA Lawlis P Duncan IJH , et al. Using time to insensibility and estimated time of death to evaluate a non penetrating captive bolt, cervical islocation, and blunt trauma for on-farm killing of turkeys. Poult Sci 2010; 89: 1345–1354. 20548061 10.3382/ps.2009-00445

[52] Gregory NG Wotton SB. Comparison of neck dislocation and percussion of the head on visual evoked responses in the chicken’s brain. Vet Rec 1990; 126: 570–572. 2368304

[53] Carbone L Carbone ET Yi EM , et al. Assessing cervical dislocation as a humane euthanasia method in mice. J Am Assoc Lab Anim Sci 2012; 51: 352–356. 22776194 PMC3358985

[54] Vanderwolf CH Buzsaki G Cain DP , et al. Neocortical and hippocampal electrical activity following decapitation in the rat. Brain Res 1988; 451: 340–344. 3251594 10.1016/0006-8993(88)90780-9

[55] Derr RF. Pain perception in decapitated rat brain. Life Sci 1991; 49: 1399–1402. 1943446 10.1016/0024-3205(91)90391-n

[56] Holson RR. Euthanasia by decapitation: evidence that this technique produces prompt, painless unconsciousness in laboratory rodents. Neurotoxicol Teratol 1992; 14: 253–257. 1522830 10.1016/0892-0362(92)90004-t

[57] Bates G. Humane issues surrounding decapitation reconsidered. J Am Vet Med Assoc 2010; 237: 1024–1028. 21034337 10.2460/javma.237.9.1024

[58] van Rijn CM Krijnen H Menting-Hermeling S , et al. Decapitation in rats: latency to unconsciousness and the “wave of death.” PLoS One 2011; 6: e16514. 21304584 10.1371/journal.pone.0016514 PMC3029360

[59] Cartner SC Barlow SC Ness TJ. Loss of cortical function in mice after decapitation, cervical dislocation, potassium chloride injection, and CO_2_ inhalation. Comp Med 2007; 57: 570–573. 18246869

[60] Keller G. Physical euthanasia methods. Lab Anim 1982; 11: 20–26.

[61] Dumont R Okonkwo D Verma S , et al. Acute spinal cord injury, part I: pathophysiologic mechanism. Clin Neuropharmacol 2001; 24: 254–264. 11586110 10.1097/00002826-200109000-00002

[62] Freeman LW Wright TW. Experimental observations of concussion and contusion of the spinal cord. Ann Surg 1953; 137: 433–443. 13031478 10.1097/00000658-195304000-00001 PMC1802525

[63] Martin JE Sandercock DA Sandilands V , et al. Welfare risks of repeated application of on-farm killing methods for poultry. Animals (Basel) 2018; 8: 39. 29543779 10.3390/ani8030039 PMC5867527

[64] Mikeska JA Klemm WR. EEG evaluation of humaneness of asphyxia and decapitation euthanasia of the laboratory rat. Lab Anim Sci 1975; 25: 175–179. 1169659

[65] Dumont R Verma S Okonkwo D , et al. Acute spinal cord injury, part II: contemporary pharmacotherapy. Clin Neuropharmacol 2001; 24: 265–279. 11586111 10.1097/00002826-200109000-00003

[66] Martin J McKeegan D Sparrey J , et al. Comparison of novel mechanical cervical dislocation and a modified captive bolt for on-farm killing of poultry on behavioural reflex responses and anatomical pathology. Anim Welf 2016; 25: 227–241.

[67] Martin JE Sandilands V Sparrey J , et al. Welfare assessment of novel on-farm killing methods for poultry. PLoS One 2019; 14: e0212872. 30794690 10.1371/journal.pone.0212872 PMC6386380

[68] Martin JE Sandilands V Sparrey J , et al. On farm evaluation of a novel mechanical cervical dislocation device for poultry. Animals (Basel) 2018; 8: 10. 29320399 10.3390/ani8010010 PMC5789305

[69] Rossi GF Zirondoli A. On the mechanism of the cortical desynchronization elicited by volatile anesthetics. Electroencephalogr Clin Neurophysiol 1955; 7: 383–390. 13251180 10.1016/0013-4694(55)90010-9

[70] Schlag J Brand H. An analysis of electrophysiological events in cerebral structures during ether anesthesia. Electroencephalogr Clin Neurophysiol 1958; 10: 305–324. 13548077 10.1016/0013-4694(58)90039-7

[71] Lee UC Blain-Moraes S Mashour GA. Assessing levels of consciousness with symbolic analysis. Philos Trans A Math Phys Eng Sci 2015; 373: 20140117. 25548273 10.1098/rsta.2014.0117 PMC7398453

[72] Boivin GP Hickman DL Creamer-Hente MA , et al. Review of CO_2_ as a euthanasia agent for laboratory rats and mice. J Am Assoc Lab Anim Sci 2017; 56: 491–499. 28903819 PMC5605172

[73] Zandt B-J ten Haken B van Dijk JG , et al. Neural dynamics during anoxia and the “wave of death.” PLoS One 2011; 6: e22127. 21779384 10.1371/journal.pone.0022127 PMC3135620

[74] Gregory N Shaw F. Penetrating captive bolt stunning and exsanguination of cattle in abattoirs. J Appl Anim Welf Sci 2000; 3: 215–230.

[75] Grandin T. Mechanical, electrical and anesthetic stunning methods for livestock. Int J Study Anim Problem 1980; 1: 242–263.

[76] Humane Slaughter Association. *Practical slaughter of poultry*, 2nd ed. Wheathampstead, UK: HSA & CJA, 2013.

[77] Steiner AR Flammer SA Beausoleil NJ , et al. Humanely ending the life of animals: research priorities to identify alternatives to carbon dioxide. Animals (Basel) 2019; 9: 911. 31684044 10.3390/ani9110911 PMC6912382

[78] Gibson TJ Rebelo CB Gowers TA , et al. Electroencephalographic assessment of concussive non-penetrative captive bolt stunning of turkeys. Br Poult Sci 2018; 59: 13–20. 29099622 10.1080/00071668.2017.1401215

[79] Cors JC Gruber AD Gunther R , et al. Electroencephalographic evaluation of the effectiveness of blunt trauma to induce loss of consciousness for on-farm killing of chickens and Turkeys. Poult Sci 2015; 94: 147–155. 25609692 10.3382/ps/peu038

[80] Raj ABM O’Callaghan M. Evaluation of a pneumatically operated captive bolt for stunning/killing broiler chickens. Br Poult Sci 2001; 42: 295–299. 11469546 10.1080/00071660120055232

[81] Cohen S Kwok M Huang J. Humane euthanasia of guinea pigs (*Cavia porcellus*) with a penetrating spring-loaded captive bolt. Animals (Basel) 2020; 10: 1356. 32764350 10.3390/ani10081356 PMC7459477

[82] Woolcott CR Torrey S Turner PV , et al. Evaluation of two models of non-penetrating captive bolt devices for on-farm euthanasia of turkeys. Animals (Basel) 2018; 8(3):42. 29558419 10.3390/ani8030042 PMC5867530

[83] Bandara RMAS Torrey S Turner PV , et al. Anatomical pathology, behavioral, and physiological responses induced by application of non-penetrating captive bolt devices in layer chickens. Front Vet Sci 2019; 6: 89. 30984770 10.3389/fvets.2019.00089 PMC6447681

[84] Hawkins P Playle L Golledge H , et al. Newcastle consensus meeting on carbon dioxide euthanasia of laboratory animals, https://www.nc3rs.org.uk/3rs-resources/euthanasia (2006, accessed 14 February, 2020).

[85] Valentim AM Guedes SR Pereira AM , et al. Euthanasia using gaseous agents in laboratory rodents. Lab Anim 2016; 50(4):241–53. 26609130 10.1177/0023677215618618

[86] Conlee KM Stephens ML Rowan AN , et al. Carbon dioxide for euthanasia: concerns regarding pain and distress, with special reference to mice and rats. Lab Anim 2005; 39: 137–161. 15901358 10.1258/0023677053739747

[87] Hackbarth H Küppers N Bohnet W. Euthanasia of rats with carbon dioxide—animal welfare aspects. Lab Anim 2000; 34: 91–96. 10759372 10.1258/002367700780578055

[88] Dripps RD Comroe JH. The respiratory and circulatory response of normal man to inhalation of 7.6 and 10.4 per cent CO_2_ with a comparison of the maximal ventilation produced by severe muscular exercise, inhalation of CO_2_ and maximal voluntary hyperventilation. Am J Physiol 1947; 149: 43–51. 20291946 10.1152/ajplegacy.1947.149.1.43

[89] Smith W Harrap SB. Behavioural and cardiovascular responses of rats to euthanasia using carbon dioxide gas. Lab Anim 1997; 31: 337–346. 9350705 10.1258/002367797780596130

[90] Krohn TC Hansen AK Dragsted N. The impact of low levels of carbon dioxide on rats. Lab Anim 2003; 37: 94–99. 12689419 10.1258/00236770360563723

[91] Beausoleil NJ Mellor DJ. Introducing breathlessness as a significant animal welfare issue. N Z Vet J 2015; 63: 41–51. 10.1080/00480169.2014.940410 25004795

[92] Turner PV Hickman DL van Luijk J , et al. Welfare impact of carbon dioxide euthanasia on laboratory mice and rats: a systematic review. Front Vet Sci 2020; 7: 411. 32793645 10.3389/fvets.2020.00411 PMC7387666

[93] Danneman PJ Stein S Walshaw SO. Humane and practical implications of using carbon dioxide mixed with oxygen for anesthesia or euthanasia of rats. Lab Anim Sci 1997; 47: 376–385. 9306311

[94] Anton F Euchner I Handwerker HO. Psychophysical examination of pain induced by defined CO_2_ pulses applied to the nasal mucosa. Pain 1992; 49: 53–60. 1594282 10.1016/0304-3959(92)90187-G

[95] Peppel P Anton F. Responses of rat medullary dorsal horn neurons following intranasal noxious chemical stimulation: effects of stimulus intensity, duration, and interstimulus interval. J Neurophysiol 1993; 70:2260–2275. 8120581 10.1152/jn.1993.70.6.2260

[96] Thürauf N Friedel I Hummel C , et al. The mucosal potential elicited by noxious chemical stimuli with CO_2_ in rats: is it a peripheral nociceptive event? Neurosci Lett 1991; 128: 297–300. 1945051 10.1016/0304-3940(91)90283-y

[97] Hummel T Mohammadian P Marchl R , et al. Pain in the trigeminal system: irritation of the nasal mucosa using short- and long-lasting stimuli. Int J Psychophysiol 2003; 47: 147–158. 12568945 10.1016/s0167-8760(02)00150-2

[98] Leach MC Bowell VA Allan TF , et al. Degrees of aversion shown by rats and mice to different concentrations of inhalational anaesthetics. Vet Rec 2002; 150: 808–815. 12120924 10.1136/vr.150.26.808

[99] Leach MC Bowell VA Allan TF , et al. Erratum: Aversion to gaseous euthanasia agents in rats and mice (Comparative Medicine 52:3 (254)). Comp Med 2002; 52: 572. 12102571

[100] Tsigos C Kyrou I Kassi E , et al. Stress, endocrine physiology and pathophysiology. In: Feingold KR et al. (eds) *Endotext [Internet]*. South Dartmouth (MA): MDText.com, 2000, 25905226.

[101] Coenen AM Drinkenburg WH Hoenderken R , et al. Carbon dioxide euthanasia in rats: oxygen supplementation minimizes signs of agitation and asphyxia. Lab Anim 1995; 29: 262–268. 7564209 10.1258/002367795781088289

[102] Hewett TA Kovacs MS Artwohl JE , et al. A comparison of euthanasia methods in rats, using carbon dioxide in prefilled and fixed flow rate filled chambers. Lab Anim Sci 1993; 43: 579–582. 8158983

[103] Altholtz LY Fowler KA Badura LL , et al. Comparison of the stress response in rats to repeated isoflurane or CO_2_:O_2_ anesthesia used for restraint during serial blood collection via the jugular vein. J Am Assoc Lab Anim Sci 2006; 45: 17–22. 16642965

[104] Barbaccia ML Roscetti G Trabucchi M , et al. Time-dependent changes in rat brain neuroactive steroid concentrations and GABAA receptor function after acute stress. Neuroendocrinology 1996; 63: 166–172. 9053781 10.1159/000126953

[105] Paul ES Harding EJ Mendl M. Measuring emotional processes in animals: the utility of a cognitive approach. Neurosci Biobehav Rev 2005; 29: 469–491. 15820551 10.1016/j.neubiorev.2005.01.002

[106] Burkholder TH Niel L Weed JL , et al. Comparison of carbon dioxide and argon euthanasia: Effects on behavior, heart rate, and respiratory lesions in rats. J Am Assoc Lab Anim Sci 2010; 49: 448–453. 20819391 PMC2919185

[107] Thomas AA Flecknell PA Golledge HDR. Combining nitrous oxide with carbon dioxide decreases the time to loss of consciousness during euthanasia in mice—refinement of animal welfare? PLoS One 2012; 7: e32290. 22438874 10.1371/journal.pone.0032290 PMC3305278

[108] Niel L Kirkden RD Weary DM. Effects of novelty on rats’ responses to CO2 exposure. Appl Anim Behav Sci 2008; 111: 183–194.

[109] Guedes SR Valentim AM Antunes LM. Mice aversion to sevoflurane, isoflurane and carbon dioxide using an approach-avoidance task. Appl Anim Behav Sci 2017; 189: 91–97.

[110] Améndola L Weary DM. Evidence for consistent individual differences in rat sensitivity to carbon dioxide. PLoS One 2019; 14: e0215808. 31017958 10.1371/journal.pone.0215808 PMC6481838

[111] Wong D Makowska IJ Weary DM. Rat aversion to isoflurane versus carbon dioxide. Biol Lett 2013; 9: 20121000. 23256183 10.1098/rsbl.2012.1000 PMC3565521

[112] Kirkden RD Niel L Lee G , et al. The validity of using an approach-avoidance test to measure the strength of aversion to carbon dioxide in rats. Appl Anim Behav Sci 2008; 114: 216–234.

[113] Boulanger Bertolus J Nemeth G , et al. Rat aversion to sevoflurane and isoflurane. Appl Anim Behav Sci 2015; 164: 73–80.

[114] Niel L Weary DM. Rats avoid exposure to carbon dioxide and argon. Appl Anim Behav Sci 2007; 107: 100–109.

[115] Boivin GP Bottomley MA Schiml PA , et al. Physiologic, behavioral, and histologic responses to various euthanasia methods in c57bl/6ntac male mice. J Am Assoc Lab Anim Sci 2017; 56: 69–78. 28905718 PMC5250498

[116] Liotti M Brannan S Egan G , et al. Brain responses associated with consciousness of breathlessness (air hunger). Proc Natl Acad Sci USA 2001; 98: 2035–2040. 11172071 10.1073/pnas.98.4.2035 PMC29377

[117] Banzett RB Lansing RW Binks AP. Air hunger: a primal sensation and a primary element of dyspnea. Compr Physiol 2021; 11: 1449–1483. 33577128 10.1002/cphy.c200001 PMC10986303

[118] Leary S Underwood W Lilly E , et al. *AVMA Guidelines for euthanasia of animals: 2013 edition*. Schaumburg, Illinois: American Veterinary Medical Association, 2013.

[119] Boivin GP Bottomley MA Dudley ES , et al. The AVMA guidelines for the euthanasia of animals provides recommendations for the use of CO_2_ for rodent euthanasia. Physiological, behavioral, and histological responses of male C57BL/6N mice to different CO_2_ chamber replacement rates. J Am Assoc Lab Anim Sci 2016; 55: 451–461. 27423153 PMC4943617

[120] Beaver B Reed W Leary S McKiernan B , et al. Report of the AVMA panel on euthanasia. J Am Vet Med Assoc 2001; 218: 669–696. 11280396 10.2460/javma.2001.218.669

[121] Marquardt N Feja M Hünigen H , et al. Euthanasia of laboratory mice: are isoflurane and sevoflurane real alternatives to carbon dioxide? PLoS One 2018; 13: e0203793. 30199551 10.1371/journal.pone.0203793 PMC6130864

[122] Hornett T Haynes A. Comparison of carbon dioxide/air mixture and nitrogen/air mixture for euthanasia of rodents. Design of a system for inhalation euthanasia. Anim Technol 1984; 35: 93–99.

[123] Britt DP. The humaneness of carbon dioxide as an agent of euthanasia for laboratory rodents. In: *Euthanasia of unwanted, injured or diseased animals or for educational or scientific purposes*. Potters Bar: Universities Federation for Animal Welfare, 1987, pp. 19–31.

[124] Moody CM Chua B Weary DM. The effect of carbon dioxide flow rate on the euthanasia of laboratory mice. Lab Anim 2014; 48: 298–304. 25097256 10.1177/0023677214546509

[125] Clarkson JM McKeegan DEF Sparrey J , et al. Determining candidate hypobaric hypoxia profiles for humane killing of laboratory mice. Front Vet Sci 2022; 9: 834478. 35400097 10.3389/fvets.2022.834478 PMC8988232

[126] Lambooy E Spanjaard W. Euthanasia of young pigs with carbon monoxide. Vet Rec 1980; 107: 59–61. 7445365 10.1136/vr.107.3.59

[127] Simonsen HB Thordal-Christensen A Ockens N. Carbon monoxide and carbon dioxide euthanasia of cats: duration and animal behaviour. Br Vet J 1981; 137: 274–278. 6788344 10.1016/s0007-1935(17)31688-3

[128] Chalifoux A Dallaire A. Physiologic and behavioral evaluation of CO euthanasia of adult dogs. Am J Vet Res 1983; 44: 2412–2417. 6686419

[129] Makowska IJ Weary DM. Rat aversion to induction with inhalant anaesthetics. Appl Anim Behav Sci 2009; 119: 229–235.

[130] Khoo SYS Lay BPP Joya J , et al. Local anaesthetic refinement of pentobarbital euthanasia reduces abdominal writhing without affecting immunohistochemical endpoints in rats. Lab Anim 2018; 52: 152–162. 28758534 10.1177/0023677217721260

[131] Zatroch KK Knight CG Reimer JN , et al. Refinement of intraperitoneal injection of sodium pentobarbital for euthanasia in laboratory rats (*Rattus norvegicus*). BMC Vet Res 2017; 13: 4–10. 28222732 10.1186/s12917-017-0982-y PMC5320784

[132] Macdonald RL Barker JL. Different actions of anticonvulsant and anesthetic barbiturates revealed by use of cultured mammalian neurons. Science 1978; 200:775–777. 205953 10.1126/science.205953

[133] Macdonald RL Olsen RW. GABAA receptor channels. Annu Rev Neurosci 1994; 17: 569–602. 7516126 10.1146/annurev.ne.17.030194.003033

[134] Olsen RW Li GD. GABA A receptors as molecular targets of general anesthetics: identification of binding sites provides clues to allosteric modulation. Can J Anesth 2011; 58: 206–215. 21194017 10.1007/s12630-010-9429-7 PMC3033524

[135] Lester PA Moore RM Shuster KA , et al. Anesthesia and Analgesia. In: Suckow MA, Stevens KA, Wilson RP (eds) *The laboratory rabbit, guinea pig, hamster, and other rodents.* Amsterdam: Elsevier, 2012, pp. 33–56.

[136] Svendsen O Kok L Lauritzen B. Nociception after intraperitoneal injection of a sodium pentobarbitone formulation with and without lidocaine in rats quantified by expression of neuronal c-fos in the spinal cord—a preliminary study. Lab Anim 2007; 41: 197–203. 17430619 10.1258/002367707780378140

[137] Dutton JW Artwohl JE Huang X , et al. Assessment of pain associated with the injection of sodium pentobarbital in laboratory mice (*Mus musculus*). J Am Assoc Lab Anim Sci 2019; 58: 373–379. 30857577 10.30802/AALAS-JAALAS-18-000094 PMC6526499

[138] Gärtner K Büttner D Döhler K , et al. Stress response of rats to handling and experimental procedures. Lab Anim 1980; 14: 267–274. 7191933 10.1258/002367780780937454

[139] Stuart SA Robinson ESJ. Reducing the stress of drug administration: Implications for the 3Rs. Sci Rep 2015; 5: 14288. 26395864 10.1038/srep14288 PMC4585806

[140] Balcombe JP Barnard ND Sandusky C. Laboratory routines cause animal stress. Contemp Top Lab Anim Sci 2004; 43: 42–51. 15669134

[141] Valentine H Williams WO Maurer KJ. Sedation or inhalant anesthesia before euthanasia with CO_2_ does not reduce behavioral or physiologic signs of pain and stress in mice. J Am Assoc Lab Anim Sci 2012; 51: 50–57. 22330868 PMC3276966

[142] Moody CM Weary DM. Mouse aversion to isoflurane versus carbon dioxide gas. Appl Anim Behav Sci 2014; 158: 95–101.

[143] Zhang H Good DJ. Comparison of hypothalamic mRNA levels in mice euthanized by CO_2_ inhalation and focused-beam microwave irradiation. Lab Anim 2011; 40: 313–318. 10.1038/laban1011-313 22358208

[144] Small A Lea J Niemeyer D , et al. Development of a microwave stunning system for cattle 2: preliminary observations on behavioural responses and EEG. Res Vet Sci 2019; 122: 72–80. 30468879 10.1016/j.rvsc.2018.11.010

[145] McLean D Meers L Ralph J , et al. Development of a microwave energy delivery system for reversible stunning of cattle. Res Vet Sci 2017; 112: 13–17. 28107666 10.1016/j.rvsc.2016.12.010

[146] Ikarashi Y Maruyama Y Stavinoha WB. Study of the use of the microwave magnetic field for the rapid inactivation of brain enzymes. Jpn J Pharmacol 1984; 35: 371–387. 6503038 10.1254/jjp.35.371

[147] Stavinoha WB Weintraub ST Modak AT. The use of microwave heating to inactivate cholinesterase in the rat brain prior to analysis for acetylcholine. J Neurochem 1973; 20: 361–371. 4698283 10.1111/j.1471-4159.1973.tb12135.x

[148] Knieriem KM Medina MA Stavinoha WB. The levels of GABA in mouse brain following tissue inactivation by microwave irradiation. J Neurochem 1977; 28:885–886. 894296 10.1111/j.1471-4159.1977.tb10645.x

[149] EC (2010) Directive 2010/63/EU of the European Parliament and Council. Annex IV, Methods of killing animals.

